# Design and Validation of an Instrument to Measure the Communication of Bad News for Nurses

**DOI:** 10.3390/nursrep15050156

**Published:** 2025-04-30

**Authors:** Manuel González-Cabrera, Sergio Martínez-Vázquez, Antonio Hernández-Martínez, Rocío Adriana Peinado-Molina, María Antonia Díaz-Ogallar, Juan Miguel Martínez-Galiano

**Affiliations:** 1 Department of Nursing, University of Jaen, 23071 Jaen, Spain; mgonzale@ujaen.es (M.G.-C.); rpmolina@ujaen.es (R.A.P.-M.); maogalla@ujaen.es (M.A.D.-O.); jgaliano@ujaen.es (J.M.M.-G.); 2 Consortium for Biomedical Research in Epidemiology and Public Health (CIBERESP), 28029 Madrid, Spain; 3 Department of Nursing, Faculty of Nursing of Ciudad Real, University of Castilla-La Mancha, 13071 Ciudad Real, Spain; antonio.hmartinez@uclm.es

**Keywords:** communication of bad news, nursing, validation, questionnaire

## Abstract

**Background:** There is a notable lack of Communication of Bad News (CBN) training for nurses, along with the absence of validated tools to assess this. The aim of this research is to design and validate an instrument to assess the communication of bad news among nursing professionals in Spain: the “Communication of Bad News in Nursing (CBNN)” tool. **Methods:** A multilevel nursing panel of experts participated in creating CBNN. A cross-sectional study was carried out on 218 Spanish nurses. Then, an Exploratory Factor Analysis (EFA), Confirmatory Factor Analysis (CFA), and a convergent validity study were carried out with the Scale of Communication Skills in Nursing Professionals in the Spanish Environment (EHC), and a reliability study using internal consistency (Cronbach’s α) and Coefficient of Intraclass Correlation (ICC). **Results:** The KMO test gave an adequate value, and Bartlett’s sphericity test was significant. The EFA identified four components (empathy and perception; environment preparation, invitation, and strategy; information given and the act of communicating; and communication method) that explained most of the variance. A good fit was observed in the CFA for most of the evaluated indicators. CBNN correlated positively with EHC and was statistically associated with considering it necessary to be trained in CBN or degree of preparation. Cronbach’s α value was excellent. The ICC of absolute agreement after 96 h was good. **Conclusions:** The Communication of Bad News in Nursing questionnaire can be considered an effective tool for assessing the communication of bad news in nursing. It serves as a self-assessment tool for nurses to identify their strengths and areas for improvement in attitudes, knowledge, and skills regarding CBN.

## 1. Introduction

The communication process of nurses with patients is closely linked to the Communication of Bad News (CBN), with CBN considered one of the most complex and challenging tasks faced by nursing professionals. A fact that reflects the importance of this communication process is that the recipients of bad news tend to remember where, when, and how it was communicated to them [1,2,3,4]. CBN can be a difficult and aggressive task, creating emotionally unstable situations for practitioners [3,4,5,6,7,8,9,10]. The need for nursing professionals to acquire CBN competencies is therefore evident, as although the content of CBN is inevitable and important, improving communication skills, how information is conveyed, and even where it is communicated can mitigate the impact. However, the current landscape of CBN training is limited, self-directed, and lacks structure, particularly in emotionally charged environments such as palliative care, emergency departments, and oncology units [11]. This scarcity underscores the emotional, ethical, and professional challenges nurses encounter when conveying difficult information, highlighting the urgent need for comprehensive training that addresses these complexities in various clinical settings [3,4,5,6,7,8,9,10].

A variety of different structured protocols as well as other support tools to facilitate the communication of bad news: SPIKES (The SPIKES protocol is particularly effective in ensuring that communication is structured, empathetic, and responsive to the patient’s needs, allowing for a more personalized approach) [12]; BREAKS (emphasizes the significance of establishing rapport and emotional support, making it a valuable tool for nurses who spend considerable time with patients) [13]; PACE (focuses on preparation and enabling patients to cope with the news, which is crucial for nurses who must often provide immediate support and guidance) [14]; and ABCDEF Framework (holistic, emphasizing the continuous nature of support and communication, which is aligned with nursing practice) [15]. Moreover, structured protocols like these serve as foundational tools for delivering bad news. However, the CBN questionnaire provides a mechanism for continuous improvement and skills development, ensuring that nurses are equipped to handle the emotional and psychological complexities involved in these conversations. Furthermore, there is no validated instrument to assess CBN skills, attitudes, and knowledge [4,6,7,8,9,10]. Nurses, in their daily work, are faced with the difficult and frequent task of CBN [6,7,8,9,10,11,12]; however, they do not have this type of tool to provide guidance, support, and evaluation in this process [16]. In the existing literature, it is noted that most clinical practice guidelines on CBN are aimed at physicians and usually in the specific field of oncology [3,12,17,18]. No instrument aimed at nurses has been identified in the literature, despite nurses being the healthcare professionals who have the most direct contact with the patient. The current training for communication in nursing regarding bad news especially in high-stress and high-pressure environments and with patients in more critical situations or with chronic and/or terminal conditions, such as in palliative care, intensive care units, emergency services, and oncology units, among others, is often limited and unstructured. This lack of training highlights the emotional, ethical, and professional challenges nurses face when delivering difficult news. To address these issues, there is an urgent need for comprehensive training that equips nurses with the necessary skills. The existence of an instrument in which nurses can self-assess their CBN training would allow them to detect their CBN training needs. Therefore, the aim is to design a valid and reliable tool to assess the knowledge, attitudes, and skills in the communication of bad news in nursing professionals.

## 2. Materials and Methods

### 2.1. Questionnaire Design and Development

In the development of the Communication of Bad News in Nursing (CBNN) instrument, we aimed to create a comprehensive questionnaire, so an extensive literature review across various national and international databases, including Scopus, Cuiden Plus, Web of Science (WOS), ENFISPO, LILACS, CINAHL, and PUBMED, was conducted. We utilized DeCS and MESH descriptors, strategically combining them with Boolean operators to refine our search. Our focus was on identifying key study variables related to the skills, knowledge, and attitudes necessary for effectively communicating bad news, which informed the development of our questionnaire items. Items for a draft questionnaire were developed based on recommendations from 10 experts hailing from diverse backgrounds, including nursing, medicine, and psychology, each with varying contexts of clinical experience and qualifications. In addition to the scoring template with the scores assigned to the different items in terms of writing, comprehension, relevance, and overall assessment, an open-ended question was included so the expert could make observations on each specific item and on the questionnaire in general. To ensure a comprehensive representation and minimize bias, a purposive sampling method was employed, guaranteeing that professionals from different profiles and regions were included. The questionnaire underwent two rounds of evaluation; in the first round, modifications were made according to expert feedback, leading to the creation of version 1. In the second round, the experts reached a unanimous consensus and approved the final text. Following this, the questionnaire was administered in a pilot study to a sample of 74 nurses working in critical and emergency care, and the initial format was retained after analyzing feedback from the participants.

### 2.2. Validation Study Design and Subject Selection

A cross-sectional descriptive observational study was conducted with nurses in 2024 in Spain. The inclusion criteria for our study required participants to be practicing registered nurses actively engaged in clinical settings. We specifically excluded individuals who were not in direct practice, such as researchers or managers, as their experiences may not accurately reflect the realities of bedside care. This focus ensures that our sample represents a diverse range of care contexts, recognizing that the skills required for communicating bad news can vary significantly across different clinical environments, including oncology, palliative care, emergency medicine, and intensive care, among others. By capturing the experiences of nurses in these varied settings, we aim to provide a comprehensive understanding of effective communication strategies in challenging situations.

A suitable sample size was estimated following the recommendations of some authors, who propose between 5 and 10 participants per item, although other authors recommend between 2 and 3, or even between 4 and 10, with the minimum number of participants being no less than 200 [19,20,21,22]. In the end, 218 nurses participated.

### 2.3. Sources of Information

A purpose-designed questionnaire was used to collect information about the participating nurses, which was previously piloted on 20 nurses of different ages, sex, work departments, and educational levels. This questionnaire did not require any modification, being the questionnaire in which information about the participant is collected. This was performed independently with the piloting of the instrument, as mentioned above.

As independent variables, data related to the professional were collected, such as sex, age, place of work, type of shifts worked, type of contract, professional experience, training profile, and variables related to CBN, such as undergraduate training, postgraduate training, perception of the need for training in the communication of bad news, consideration of whether it forms part of nursing competencies, and the degree of concern and preparation for communicating bad news. As a dependent variable, the value of the questionnaire on communicating bad news in nursing was established. The EHC (Communication Skills Scale for Nursing Professionals in the Spanish Environment) was also used as an instrument to assess communication skills in different health professionals.

### 2.4. Data Analysis

A descriptive analysis was carried out using absolute frequencies for qualitative variables and means with standard deviations (SD) for quantitative variables. The validity of the questionnaire was assessed, considering content, construct, and criterion validity. For content validity, the relevance of the items was analyzed using the Content Validity Index (CVI), establishing that the values should be greater than 0.80 [23,24]. In the construct validity analysis, for this to be the case, the KMO must be above 0.6 and as close to 1 as possible, and Bartlett’s sphericity, which consists of statistical hypothesis testing, must be less than 0.05 to reject the null hypothesis of sphericity and ensure that the factor model is adequate to explain the data. In the Exploratory Factor Analysis (EFA), we use Varimax rotation to help clarify the assignment of items to different factors. To determine the number of factors we wanted to maintain, we used the Kaiser criterion, one of the most used. It retains factors with eigenvalues greater than the unit value [25].

In addition, convergent criterion validity was studied by comparing the CBNN and EHC questionnaires, using Spearman’s and Pearson’s correlation coefficients, with the expectation of obtaining a correlation >0.60. The relationship between CBNN and EHC items was also analyzed using ANOVA. Concurrent criterion validity was analyzed in relation to factors associated with the communication of bad news in nursing, using bivariate analysis significant at *p* < 0.05.

For the confirmatory factor analysis, model fits were determined through statistical values such as Chi-square and Root Mean Square Error of Approximation (RMSEA), as well as comparative fit indices (Comparative Fit Index (CFI), Tucker–Lewis Index (TLI), Normed Fit Index (NFI)) and parsimony measures (Parsimony ratio (PRATIO), Comparative Fixed Parsimony Index (PCFI), Parsimony Normed Fit Index (PNFI), Akaike Information Criterion (AIC)). Reliability was assessed using Cronbach’s alpha [26], where values are classified as excellent (α > 0.9), good (α > 0.8), acceptable (α > 0.7), questionable (α > 0.6), poor (α > 0.5), and unacceptable (α < 0.5) [27].

Finally, temporal stability was assessed by test–retest with a sample of 33 nurses, calculating the intraclass correlation coefficient (ICC) after 96 h.

### 2.5. Ethical Considerations

The study was approved by the provincial research ethics committee of Jaén (MGC17). Participants received an information sheet and signed the informed consent form.

## 3. Results

A study was conducted with 218 nursing professionals; 76.6% (167) were female, with an average age of 39.40 years (SD=12.19 years). Most of the participants, 83% (181), worked in the public sector, and 40.8% (89) had more than 10 years of experience. In terms of training in communicating bad news, 51.8% (113) received training during their undergraduate studies, and only 29.8% (65) were at the postgraduate level. 93.6% (204) consider this communication as part of nursing competencies. Although 50.9% (111) feel somewhat prepared to communicate bad news, more than half (56%, 122) are quite concerned about doing so. 26.1% (57) had never communicated bad news, while 73.9% (161) had. The characteristics of the sample can be seen in more detail in Table 1.

The mean score on the scale of communicating bad news was 103.13 (SD = 16.6) points. Regarding the participants’ responses in the CBNN questionnaire, the most chosen response options were “always” in item 28 with 78.9% (172), item 6 with 73.9% (161), item 5 with 72.9% (159), and item 13 with 71.1% (155), followed by item 4 with 67% (146). In contrast, the least marked options were “never” in items 4, 5, 6, 13, 14, 21, and 25; “almost never” in items 6, 13, and 28; and “always” in item 7, all with <1% (2) choice in each. All the participants’ response options, as well as their distribution obtained in the measurement instrument for the assessment of the communication of bad news, are shown in Table 2.

### 3.1. Validation of the Questionnaire

#### 3.1.1. Content Validity

As for content validity, the analysis of the judgments made by the experts resulted in a high level of agreement on the suitability of the items, excluding only 3 items for having median scores higher than 2, leaving the first version of the questionnaire with 28 items. The items eliminated were 9, 24, and 25 (Table 3). This questionnaire was assessed a second time by the 10 experts, and the suitability of all the items was approved, with a median score for each item between 1 and 2 points (maximum agreement).

The distribution and the different categories established in the expert assessment template are set out below. **Editorial Staff:** 1 point: Very well written; 2 points: Well written; 3 points: Acceptable; 4 points: Poorly written; 5 points: Very poorly written. **Comprehension:** 1 point: A lot of; 2 points: Quite a bit; 3 points: Regular; 4 points: A little; 5 points: Very little. **Relevance:** 1 point: Very relevant; 2 points: Fairly relevant; 3 points: Relevant; 4 points: Little relevant; 5 points: Not relevant at all. **Overall rating:** 1 point: Very good; 2 points: Good; 3 points: Regular; 4 points: Poor; 5 points: Very poor.

#### 3.1.2. Construct Validity

The KMO test showed a value of 0.925, and Bartlett’s test of sphericity was <0.001. In this analysis, it was observed in the anti-image correlation matrix that all the values were above 0.811, except for item 7, with a value of 0.494, and item 16, with a value of 0.562. It was therefore decided to exclude it. In this way, the scale now had 26 items with a KMO test value of 0.931 and Bartlett’s test of sphericity of <0.001, and all the values increased and were above 0.818, as can be seen in Table 4. Four components were obtained that explained 61.96% of the variance, which can be seen in Table 5.

The first component obtained, corresponding to perception and empathy, consisted of items 11, 12, 13, 14, 17, 21, 24, 25, and 28, which explained 44.23% of the variance. The second component, corresponding to environmental readiness, invitation, and strategy, consisted of items 1, 2, 3, 8, 9, 10, 26, and 27 and explained 7.45% of the variance. The third component, which deals with information and communication action, consisted of items 15, 18, 19, 20, 22, and 23 and accounted for 6.24% of the variance. The fourth component, consisting of the form of communication, consisted of items 4, 5, and 6, which accounted for 4.24% of the variance. These components can be seen in Table 6.

#### 3.1.3. Convergent Validity

A bivariate analysis was carried out to assess the convergent validity of the questionnaire on Communication of Bad News in Nursing (CBNN) training among nurses, finding significant relationships with gender (*p* = 0.007), where women had higher scores (104.76; SD = 16.27) than men (97.78; SD = 14.74). In terms of workplace, the public sector had the highest score (103.06; SD = 15.67), but without statistical significance. Health and social care facility nurses had the best score (109.82; SD = 12.03) compared to other areas, but without statistical significance. Day shifts showed a higher score (105.60; SD = 14.30) compared to on-call shifts (100.16; SD = 12.32), with no statistical significance between them. Regarding work experience, professionals with less than one year of experience scored higher (106.22; SD = 23.51), but no significance was found. Nurses with a doctoral degree had higher scores (110.71; SD = 10.13); however, there was no significant statistical relationship between educational levels and scores. Regarding CBN training, those who received CBN training scored 103.89 (SD = 16.10), while those who had not received CBN training scored 102.30 (SD = 16.27), with no significance. However, nurses who felt training was necessary scored 103.37 (SD = 15.79), showing a statistically positive relationship (*p* < 0.001). Those who felt better prepared for CBN scored higher, with a notable statistical relationship (*p* < 0.001) between perceived preparedness and scores. Finally, with respect to concern about CBN, no significant differences in scores were observed. Previous experience in communicating bad news also showed no significant relationship, as scores were similar between those who had and those who had not. The distribution of scores on the scale can be seen in Table 7.

#### 3.1.4. Criterion Validity

**Figure 1:**
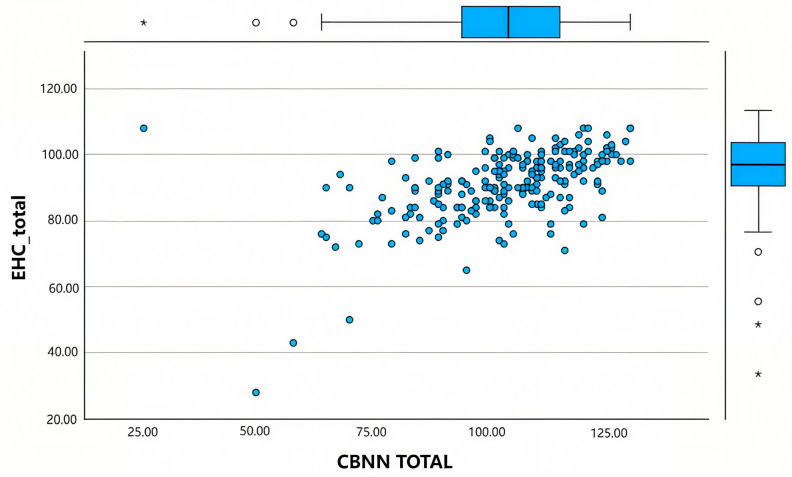
Convergent validity of CBNN with EHC.

For the criterion validity study, the CBNN questionnaire was used with the EHC scale, using Pearson’s correlation coefficient. A statistically significant and positive relationship was observed between the EHC scale and the CBNN questionnaire: r = 0.545 (95% CI: 0.44–0.63), *p* < 0.001; this can be seen in Figure 1.

### 3.2. Internal Consistency

For the internal consistency study, the α of the total questionnaire was used. Thus, for the CBNN scale, an α = 0.944 was obtained after eliminating items 7 and 16. The values for each factor are shown in Table 8.

### 3.3. Temporary Stability

To determine the temporal stability of the CBNN, a random sample of 33 nursing professionals was selected and given the questionnaire, which was re-administered 96 h later for completion. As a result of the test–retest, an absolute agreement ICC of 0.791 (95% CI 0.58–0.90) was obtained, which was considered good.

### 3.4. Confirmatory Factor Analysis

**Figure 2:**
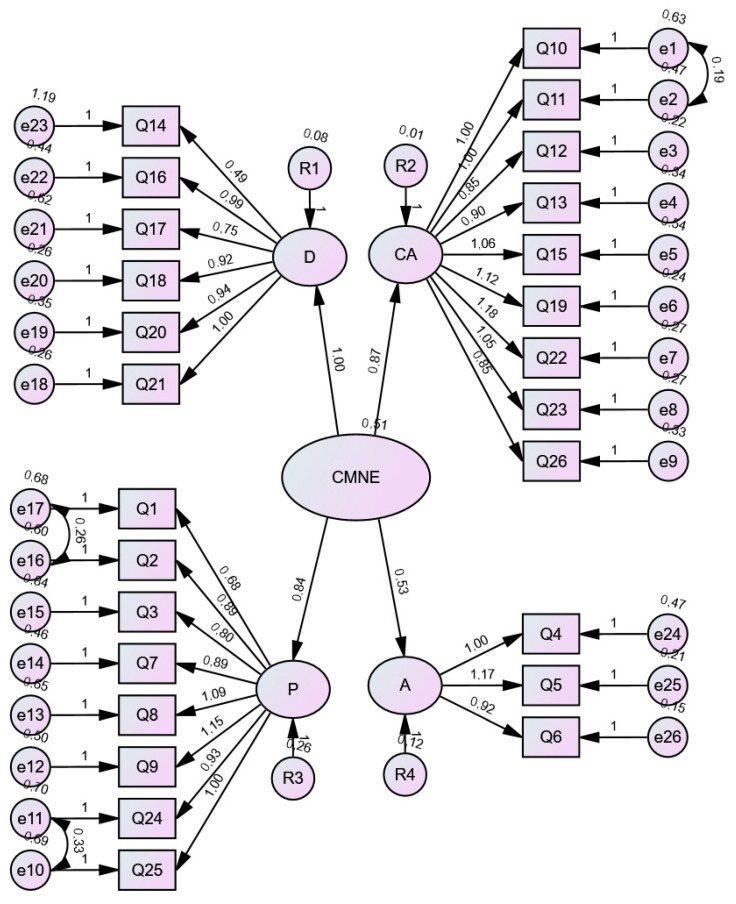
Path diagram CBNN.

After performing the confirmatory factor analysis, a good model fit was observed in the absolute RMSEA (0.079), the incremental fit indices: TLI (0.868), CFI (0.881), and NFI (0.812), and the parsimonious fit index PCFI (0.792). Table 9 shows all the values for each indicator and the criteria needed to confirm the model fit. The path diagram can be seen in Figure 2.

## 4. Discussion

The CBNN questionnaire has been rated very positively by the experts, showing adequate psychometric characteristics. Better scores on the CBNN questionnaire were associated with nurses who considered CBN training important, having had previous CBN training, and being a woman.

The CICAA questionnaire (Connect, Identify, Understand, Agree, Help), a scale to assess the clinical relationship during the care process [28], covers a broader field of clinical communication in general and not of bad news, unlike the CBNN, which is designed for a more specific situation, and thus validates more specific items related to empathy in high-stress situations, “clarity of information under adverse conditions and immediate emotional support”. The treatment, however, was very similar since our questionnaire started with 31 initial items and, after the experts’ judgment and assessment, finally resulted in 28 items [29,30]. The agreement among the experts, assessed through the Content Validity Index (CVI), showed that the selected items were relevant and representative of the appropriate theoretical dimensions, which strengthens their applicability, something highlighted by other authors in similar studies [31].

Regarding construct validity, a study [32] in which the CICAA questionnaire was replicated in 154 nursing students with a KMO value of 0.921, which after Bartlett’s test was statistically significant (*p* < 0.001), confirmed the existence of four principal components that accounted for 67.78% of the variance with the initial 29 items. This strengthens the similarity and power of our data, in line with our study’s Exploratory Factor Analysis [33].

As for convergent validity, there is no tool similar to the CBNN developed in this study, although there is already another scale that measures communication skills in healthcare professionals in Spain [34]. The main objective of this scale was to analyze its psychometric properties by establishing a scale for nursing professionals. It was applied for validation on a sample of 692 nurses, acting with an item discrimination index > 30 in its items, obtaining four factors after the CFA. Adequate psychometric properties, internal structure, reliability, and validity, along with an acceptable specificity for communication in difficult situations, were obtained.

If the results are observed, the nurses’ perception of the need for training in CBN increased the value obtained in the CBNN questionnaire, as well as the degree of training they had in communicating bad news. This can be reinforced by reviewing studies, such as the one conducted by Betancourth et al. [35], on social skills related to the communication process, which relate the perception of the need for training and their degree of preparation directly to professional competence in CBN.

In relation to internal consistency, several validated scales have been identified in the literature that show proven reliability and internal consistency according to their results [36,37]. Thus, the EHC scale, which monitors communication skills in nursing professionals [34], shows high internal consistency, with Cronbach’s alpha values of 0.88 overall and 0.70–0.77 partially for its dimensions. Similarly, the BAS scale (the Breaking Bad News Assessment Schedule) [38], which is somewhat more specific in CBN, yields a Cronbach’s α value of 0.93. In addition, the “Communication Skill Questionnaire” (CSQ), a Spanish adaptation by Prat G et al. [39] of the original by Takahashi et al. [40], shows a reliability and internal consistency with Cronbach’s alpha values of 0.960, similar to those obtained in our study [37].

Regarding temporal stability, the test–retest found an ICC of absolute agreement that was considered good. The CICAA questionnaire [28], which shows an ICC reliability of 0.967 (95% CI, 0.933–0.984), as well as the results obtained in the “Communication Skill Questionnaire” CSQ [40], show results with coefficients ranging between 0.60 and 0.70. These data obtained in these validated scales on test–retest reliability, when compared with those obtained by the CBNN, in which an absolute agreement ICC of 0.791 (95% CI 0.58–0.90) was obtained, show values considered to be good [41]. In the confirmatory factor analysis and its determination of model fit, where acceptable absolute, incremental, and parsimonious fit index values were obtained, the results obtained in the AFC of the EHC scale [34], which obtained four main factors, very similar to the four main factors obtained in the CBNN questionnaire [42], have been compared.

As this is a questionnaire, a possible selection bias associated with non-response cannot be ruled out, but the number of non-responding participants was very low at 12. However, there are no indications or reasons to suggest that the nurses who did not participate would have responded differently from those who did. In this sense, anamnestic bias cannot be completely ruled out either. The type of study and its execution could also bring limitations belonging inherently to its design. The effectiveness of the CBNN questionnaire in enhancing communication skills among nurses largely depends on its integration into nursing practice. 

As a strength, the group of experts had different professional profiles from the different areas of nursing. These experts also have extensive experience in communicating bad news. While exploring future applications of the CBNN questionnaire is essential, it is crucial to first ensure consistent training and robust support systems within nursing practice. Addressing these foundational elements will enhance its effectiveness in improving communication skills among nurses, ultimately leading to better patient care across various clinical contexts.

The CBNN questionnaire provides an effective tool for evaluating the communication of bad news for nursing professionals in terms of attitudes, knowledge, and skills. Likewise, this new questionnaire allows the assessment of attitudes, knowledge, and skills of nursing professionals in this context and serves as a self-assessment tool to identify strengths and areas for improvement. Its implementation in clinical practice aims to improve the quality of care in difficult situations of communicating bad news and opens avenues for studies in this area. This could inform the creation of specialized assessment tools and tailored educational programs aimed at improving communication skills. Such efforts may lead to better patient outcomes.

## 5. Conclusions

The Communication of Bad News in Nursing Questionnaire (CBNN) shows strong reliability and validity, with factors like gender, training needs, and preparedness enhancing scores. Overall, while the CBNN serves as a solid assessment foundation, expanding research is essential for ongoing improvement in nursing communication practices.

## Figures and Tables

**Table 1:** Sample characteristics.

Variable	n (%)
**Mean CBNN score (SD)**	103.13 (16.6)
**Biological sex**	
Male	51 (23.4)
Female	167 (76.6)
**Current workplace**	
Public sector	181 (83.0)
Private sector	14 (6.4)
Both (public and private sectors)	15 (6.9)
Not currently working	8 (3.7)
**Current position**	
Emergency	40 (18.3)
Intensive Critical Unit	16 (7.3)
Hospital care	45 (20.6)
Family nurse	33 (15.1)
Outpatients	10 (4.6)
Operating theatre	10 (4.6)
Palliative care	4 (1.8)
Socio-health Centre	11 (5.0)
Unemployed	7 (3.2)
Another	42 (19.3)
**Work shift**	
Rotational shift work	112 (51.4)
Daytime	71 (32.6)
On-call	32 (14.7)
Nights	3 (1.4)
**Type of contract (current)**	
Statutory	83 (38.1)
Interim	37 (17.0)
Eventual	75 (34.4)
Another	23 (10.6)
**Professional experience**	
<1 year	18 (8.3)
1–5 years	50 (22.9)
5–10 years	61 (28.0)
>10 years	89 (40.8)
**Education profile**	
Diploma/Graduate	115 (52.8)
Expert	9 (4.1)
Master’s degree	61 (28.0)
Specialization	26 (11.9)
PhD	7 (3.2)
**Previous undergraduate training**	
**Have you received CBN training?**	
Yes	113 (51.8)
No	105 (48.2)
**Postgraduate training**	
**Have you received CBN training?**	
Yes	65 (29.8)
No	153 (70.2)
**Do you see a need for CBN training?**	
Yes	217 (99.5)
No	1 (0.5)
**Do you consider that CBN is part of nursing work?**	
Yes	204 (93.6)
No	14 (6.4)
**Level of preparedness for CBN**	
None	13 (6.0)
Little	53 (24.3)
Some	111 (50.9)
Quite well prepared	38 (17.4)
Very prepared	3 (1.4)
**Level of concern for CBN**	
No concern	1 (0.5)
A little concern	4 (1.8)
Some concern	26 (11.9)
Quite concerned	122 (56.0)
Very concerned	65 (29.8)
**Have you ever reported bad news?**	
Yes	161 (73.9)
No	57 (26.1)
**If you have made the communication of bad news, Do you ever specify in which service?** **n (%)**	
Hospital emergency care	25 (11.5)
Out-of-hospital emergencies	11 (5.0)
Intensive Critical Unit	16 (7.3)
Palliative care	11 (5.0)
Hospital care	38 (17.4)
Primary care	14 (6.4)
Mental health	2 (0.9)
Socio-health Centre	3 (1.4)
Old people’s home	12 (5.5)
Delivery unit	5 (2.3)
Oncology	7 (3.2)
Emergency Call Center	2 (0.9)
Ambiguous	3 (1.4)
Pediatrics	1 (0.5)

**Table 2:** Scoring and distribution of CBNN.

Item	Never N (%)	Hardly Ever N (%)	Sometimes N (%)	Most of the Time N (%)	Always N (%)
**CBNN1**	5 (2.3)	12 (5.5)	48 (22.0)	82 (37.6)	71 (32.6)
**CBNN2**	10 (4.6)	19 (8.7)	55 (25.2)	89 (40.8)	45 (20.6)
**CBNN3**	35 (16.1)	71 (32.6)	63 (28.9)	36 (16.5)	13 (6.0)
**CBNN4**	2 (0.9)	7 (3.2)	20 (9.2)	43 (19.7)	146 (67.0)
**CBNN5**	2 (0.9)	4 (1.8)	13 (6.0)	40 (18.3)	159 (72.9)
**CBNN6**	1 (0.5)	1 (0.5)	8 (3.7)	47 (21.6)	161 (73.9)
**CBNN7**	123 (56.4)	61 (28.0)	19 (8.7)	13 (6.0)	2 (0.9)
**CBNN8**	3 (1.4)	14 (6.4)	48 (22.0)	75 (34.4)	78 (35.8)
**CBNN9**	16 (7.3)	35 (16.1)	63 (28.9)	60 (27.5)	44 (20.2)
**CBNN10**	10 (4.6)	43 (19.7)	58 (26.6)	62 (28.4)	45 (20.6)
**CBNN11**	4 (1.89)	17 (7.8)	39 (17.9)	77 (35.3)	81 (37.2)
**CBNN12**	3 (1.4)	7 (3.2)	33 (15.1)	57 (26.1)	118 (54.1)
**CBNN13**	1 (0.5)	1 (0.5)	20 (9.2)	41 (18.8)	155 (71.1)
**CBNN14**	2 (0.9)	3 (1.4)	25 (11.5)	72 (33.0)	116 (53.2)
**CBNN15**	21 (9.6)	48 (22.0)	65 (29.8)	59 (27.1)	25 (11.5)
**CBNN16**	39(17.9)	66 (30.3)	77 (35.3)	27 (12.4)	9 (4.1)
**CBNN17**	5 (2.3)	24 (11.0)	64 (29.4)	82 (37.6)	43 (19.7)
**CBNN18**	4 (1.8)	20 (9.2)	45 (20.6)	83 (38.1)	66 (30.3)
**CBNN19**	5 (2.3)	17 (7.8)	54 (24.8)	89 (40.8)	53 (24.3)
**CBNN20**	3 (1.4)	5 (2.3)	26 (11.9)	73 (33.5)	111 (50.9)
**CBNN21**	2 (0.9)	7 (3.2)	23 (10.6)	70 (32.1)	116 (53.2)
**CBNN22**	3 (1.4)	12 (5.5)	41 (18.8)	89 (40.8)	73 33.5)
**CBNN23**	4 (1.8)	10 (4.6)	51 (18.8)	92 (42.2)	61 (28.0)
**CBNN24**	3 (1.4)	8 (3.7)	29 (13.3)	78 (35.8)	100 (45.9)
**CBNN25**	2 (0.9)	6 (2.8)	26 (11.9)	84 (38.5)	100 (45.9)
**CBNN26**	7 (3.2)	35 (16.1)	47 (21.6)	75 (34.4)	54 (24.8)
**CBNN27**	9 (4.1)	30 (13.8)	64 (29.4)	55 (25.2)	60 (27.5)
**CBNN28**	4 (1.8)	0 (0.0)	18 (8.3)	24 (11.0)	172 (78.9)

**Table 3:** Evaluation of items by experts.

Elements	Editorial Staff	Comprehension	Relevance	Overall
	Median (IQR)	Median (IQR)	Median (IQR)	Median (IQR)
**Item 1**	1.5 (1)	1.0 (1)	1.0 (1)	1.0 (1)
**Item 2**	1.0 (1)	1.0 (1)	1.0 (1)	1.0 (1)
**Item 3**	2.0 (2)	2.0 (2)	2.0 (3)	2.0 (2)
**Item 4**	1.0 (1)	1.0 (1)	1.0 (1)	1.0 (1)
**Item 5**	1.0 (1)	1.0 (1)	1.0 (1)	1.0 (1)
**Item 6**	1.0 (1)	1.0 (1)	1.0 (1)	1.0 (1)
**Item 7**	2.0 (2)	1.5 (1)	1.0 (1)	1.5 (2)
**Item 8**	2.0 (2)	2.0 (2)	1.0 (1)	2.0 (2)
**Item 9**	3.0 (1)	3.0 (1)	1.5 (1)	2.0 (2)
**Item 10**	2.5 (2)	2.0 (2)	2.0 (3)	2.5 (2)
**Item 11**	1.0 (1)	1.0 (1)	2.5 (3)	1.5 (3)
**Item 12**	2.0 (3)	1.5 (1)	1.0 (1)	2.0 (2)
**Item 13**	2.0 (3)	1.5 (2)	2.0 (2)	1.5 (2)
**Item 14**	1.5 (2)	1.0 (1)	1.0 (1)	1.0 (1)
**Item 15**	2.5 (2)	2.0 (1)	1.0 (1)	1.5 (1)
**Item 16**	2.0 (2)	1.5 (1)	2.0 (2)	1.5 (2)
**Item 17**	1.5 (1)	1.0 (1)	1.5 (3)	1.0 (1)
**Item 18**	2.0 (2)	2.0 (1)	1.5 (2)	2.0 (1)
**Item 19**	1.5 (2)	1.5 (1)	1.0 (1)	1.5 (1)
**Item 20**	1.5 (2)	1.0 (1)	1.0 (1)	1.0 (1)
**Item 21**	2.0 (1)	1.5 (2)	1.5 (2)	1.5 (1)
**Item 22**	2.0 (1)	2.0 (0)	2.0 (1)	2.0 (1)
**Item 23**	1.5 (1)	2.0 (2)	1.0 (1)	1.0 (1)
**Item 24**	2.0 (2)	3.0 (1)	2.0 (2)	2.0 (2)
**Item 25**	2.5 (3)	2.5 (3)	2.0 (2)	2.5 (2)
**Item 26**	1.5 (1)	1.5 (2)	1.0 (1)	1.0 (1)
**Item 27**	2.0 (1)	2.0 (2)	1.0 (1)	1.5 (1)
**Item 28**	1.0 (1)	2.0 (2)	1.0 (2)	1.5 (2)
**Item 29**	1.5 (2)	1.5 (1)	1.5 (1)	1.5 (1)
**Item 30**	2.0 (3)	2.0 (2)	1.0 (1)	1.0 (1)
**Item 31**	1.0 (1)	1.0 (1)	1.0 (1)	1.0 (1)

IQR: Interquartile range.

**Table 4:** Anti-image correlation matrix.

28 ITEMS		AFTER REMOVING 7 & 16	
**IMCBNN 1**	0.911	**IMCBNN 1**	0.914
**IMCBNN 2**	0.900	**IMCBNN 2**	0.908
**IMCBNN 3**	0914	**IMCBNN 3**	0.913
**IMCBNN 4**	0.941	**IMCBNN 4**	0.941
**IMCBNN 5**	0.897	**IMCBNN 5**	0.895
**IMCBNN 6**	0.927	**IMCBNN 6**	0.928
**IMCBNN 7**	0.494	**IMCBNN 8**	0.920
**IMCBNN 8**	0.911	**IMCBNN 9**	0.949
**IMCBNN 9**	0.948	**IMCBNN 10**	0.907
**IMCBNN 10**	0.895	**IMCBNN 11**	0.943
**IMCBNN 11**	0.945	**IMCBNN 12**	0.948
**IMCBNN 12**	0.946	**IMCBNN 13**	0.940
**IMCBNN 13**	0.936	**IMCBNN 14**	0.928
**IMCBNN 14**	0.930	**IMCBNN 15**	0.818
**IMCBNN 15**	0.811	**IMCBNN 17**	0.931
**IMCBNN 16**	0.562	**IMCBNN 18**	0.926
**IMCBNN 17**	0.929	**IMCBNN 19**	0.928
**IMCBNN 18**	0.925	**IMCBNN 20**	0.925
**IMCBNN 19**	0.933	**IMCBNN 21**	0.937
**IMCBNN 20**	0.924	**IMCBNN 22**	0.952
**IMCBNN 21**	0.938	**IMCBNN 23**	0.960
**IMCBNN 22**	0.951	**IMCBNN 24**	0.972
**IMCBNN 23**	0.959	**IMCBNN 25**	0.954
**IMCBNN 24**	0.969	**IMCBNN 26**	0.894
**IMCBNN 25**	0.956	**IMCBNN 27**	0.905
**IMCBNN 26**	0.889	**IMCBNN 28**	0.945
**IMCBNN 27**	0.894		
**IMCBNN 28**	0.940		

**Table 5:** Total variance explained.

Components	Initial Values			Rotational Sums of Extracted Squared			Rotational Sums of Squared Loads		
	Total	% De Variance	% Accumulated	Total	% De Variance	% Accumulated	Total	% De Variance	% Accumulated
**1**	11.499	44.227	44.227	11.499	44.227	44.227	5.171	19.890	19.890
**2**	1.937	7.450	51.677	1.937	7.450	51.627	4.457	17.142	37.032
**3**	1.624	6.244	57.922	1.624	6.244	57.922	3.703	14.242	51.274
**4**	1.049	4.037	61.958	1.049	4.037	61.958	2.778	10.684	63.958
**5**	1.020	3.925	65.883						
**6**	0.847	3.259	69.142						
**7**	0.803	3.089	72.231						
**8**	0.692	2.660	74.891						
**9**	0.632	2.429	77.321						
**10**	0.570	2.191	79.511						
**11**	0.528	2.032	81.543						
**12**	0.500	1.923	83.466						
**13**	0.481	1.850	85.316						
**14**	0.473	1.818	87.134						
**15**	0.392	1.509	88.643						
**16**	0.385	1.479	90.122						
**17**	0.342	1.316	91.438						
**18**	0.335	1.290	92.728						
**19**	0.321	1.235	93.963						
**20**	0.288	1.106	95.070						
**21**	0.278	1.068	96.138						
**22**	0.249	0.957	97.095						
**23**	0.232	0.893	97.988						
**24**	0.203	0.781	98.768						
**25**	0.180	0.692	99.460						
**26**	0.140	0.540	100.000						

Extraction method. Principal component analysis.

**Table 6:** Rotated component matrix. Principal component analysis.

	Components			
Items	Empathy and Perception	Environment Preparation, Invitation and Strategy	Information Given and the Act of Communicate	Communication Method
**CBNN1**		0.499		
**CBNN 2**		0.677		
**CBNN 3**		0.713		
**CBNN 4**				0.684
**CBNN 5**				0.749
**CBNN 6**				0.703
**CBNN 8**		0.556		
**CBNN 9**		0.709		
**CBNN 10**		0.747		
**CBNN 11**	0.496			
**CBNN 12**	0.649			
**CBNN 13**	0.654			
**CBNN 14**	0.731			
**CBNN 15**			0.523	
**CBNN 17**	0.497			
**CBNN 18**			0.606	
**CBNN 19**			0.758	
**CBNN 20**			0.600	
**CBNN 21**	0.608		0.583	
**CBNN 22**			0.566	
**CBNN 23**			0.535	
**CBNN 24**	0.599			
**CBNN 25**	0.628			
**CBNN 26**		0.552		
**CBNN 27**		0.624		
**CBNN 28**	0.552			

Rotation method. Varimax and Kaiser normalisation.

**Table 7:** Mean CBNN score and interrelationship.

Variable	CBNN Scoring	*p* Value (Combined)
	**(SD)**	
**CBNN**	103.13 (16.16)	
**EHC**	90.81 (10.54)	
**Age**	39.40 (12.19)	
**Biological sex**		**0.007**
Male	97.78 (14.74)	
Female	104.76 (16.27)	
**Current workplace**		0.989
Public	103.06 (15.67)	
Private	102.79 (15.59)	
Both	104.53 (12.10)	
Not currently working	102.75 (32.23)	
**Current position**		0.277
Emergency	98.07 (12.99)	
ICU	104.87 (19.06	
Hospital care	100.51 (19.03)	
Family Nurse	106.88 (11.81)	
Consultations	107.10 (15.49)	
Operating theatre	104.60 (10.99)	
Palliative care	104.25 (23.64)	
Socio-health centre	109.82 (12.03)	
Unemployed	97.57 (34.02)	
Other	104.90 (14.04)	
**Type of shift pattern**		0.384
Rotational shift work	102.35 (18.17)	
Daytime	105.60 (14.30)	
24 h shifts	100.16 (12.32)	
Night	105.33 (10.41)	
**Type of contract (current)**		0.295
Statutory	101.14 (17.15)	
Interim	107.27 (13.84)	
Eventual	103.41 (13.95)	
Another	102.69 (21.63)	
**Professional experience**		0.550
<1 year	106.22 (23.51)	
1–5 years	104.74 (14.29)	
5–10 years	101.10 (13.07)	
>10 years	102.98 (17.35)	
**Educational profile**		0.776
Diploma/Graduate	102.75 (18.14)	
Expert	105.11 (17.29	
Master’s degree	102.64 (12.45)	
Specialization	103.23 (15.99)	
PhD	110.71 (10.13)	
**Previous undergraduate training** **Have you received CBN training?**		0.470
Yes	103.89 (16.10)	
No	102.30 (16.27)	
**Postgraduate training** **Have you received CBN training?**		0.211
Yes	105.23 (15.86)	
No	102.23 (16.26)	
**Do you see a need for CBN training?**		**0.001**
Yes	103.37 (15.79)	
No	50.00	
**Do you consider that CBN is a part of nursing work?**		0.095
Yes	103.61 (15.79)	
No	96.14 (20.29)	
**Degree of preparation for CBN**		**<0.001**
None	87.77 (25.81)	
Little	100.90 (14.23)	
Some	103.21 (14.98)	
OK	111.16 (13.75)	
A lot	104.33 (18.58)	
**Degree of concern about CBN**		0.923
No concern	109.00	
A little concerned	98.00 (11.04)	
Some concern	105.04 (11.35)	
Quite concerned	102.93 (17.01)	
Very concerned	102.97 (16.71)	
**Have you ever given bad news?**		0.573
Yes	103.50 (15.38)	
No	102.09 (18.31)	

Statistically significant values in bold.

**Table 8:** Reliability of the CBNN questionnaire.

Reliability with All Items of the CBNN Questionnaire		Reliability When Items 7 and 16 of the CBNN Questionnaire Are Removed	
CBNN	Cronbach’Alpha (α)	CBNN	Cronbach’Alpha (α)
**Total**	0.935	**Total**	0.944
**CBNN1**	0.933	**CBNN 1**	0.943
**CBNN 2**	0.932	**CBNN 2**	0.942
**CBNN 3**	0.934	**CBNN 3**	0.944
**CBNN 4**	0.934	**CBNN 4**	0.944
**CBNN 5**	0.933	**CBNN 5**	0.943
**CBNN 6**	0.933	**CBNN 6**	0.943
**CBNN 7**	0.940	**CBNN 8**	0.942
**CBNN 8**	0.932	**CBNN 9**	0.943
**CBNN 9**	0.932	**CBNN 10**	0.942
**CBNN 10**	0.931	**CBNN 11**	0.942
**CBNN 11**	0.932	**CBNN 12**	0.942
**CBNN 12**	0.932	**CBNN 13**	0.942
**CBNN 13**	0.932	**CBNN 14**	0.942
**CBNN 14**	0.932	**CBNN 15**	0.947
**CBNN 15**	0.936	**CBNN 17**	0.942
**CBNN16**	0.939	**CBNN 18**	0.941
**CBNN 17**	0.931	**CBNN 19**	0.944
**CBNN 18**	0.931	**CBNN 20**	0.941
**CBNN 19**	0.933	**CBNN 21**	0.941
**CBNN20**	0.931	**CBNN 22**	0.941
**CBNN 21**	0.931	**CBNN 23**	0.941
**CBNN 22**	0.931	**CBNN 24**	0.940
**CBNN 23**	0.930	**CBNN 25**	0.941
**CBNN24**	0.930	**CBNN 26**	0.942
**CBNN 25**	0.931	**CBNN 27**	0.942
**CBNN 26**	0.931	**CBNN 28**	0.943
**CBNN 27**	0.932		
**CBNN 28**	0.932		

**Table 9:** Confirmatory Factor Analysis. Values of the original model and values after error correlation.

Indicators	Benchpoints	Estimated Values After Correlating Errors
**Absolute adjustment indices**		
**Chi-square**	>0.005	**<0.001**
**Root Mean Square Error of Approximation (RMSEA)**	<0.08	**0.079**
**Incremental Adjustment Indices**		
**Tucker–Lewis Index (TLI)**	>0.90	**0.868**
**Comparative Fit Index (CFI)**	>0.90	**0.881**
**Normed Fit Index (NFI)**	>0.90	**0.812**
**Parsimonious Adjustment Indices**		
**Parsimony Ratio (PRATIO)**	>0.90	0.898
**Comparative Fixed Parsimony Index (PCFI)**	>0.80	**0.792**
**Parsimony Normed fit Index (PNFI)**	>0.80	0.729
**Akaike Information Criterion (AIC)**	Minor value	803.960

Bold: Acceptable fit criteria for confirmatory factor analysis.

## Data Availability

The data are available from the authors upon request via email.

## Public Involvement Statement

There was no public involvement in any aspect of this research.

## Guidelines and Standards Statement

This manuscript was drafted against the STROBE guidelines.
